# Application of a Mechanistic Model for the Prediction of Microcystin Production by *Microcystis* in Lab Cultures and Tropical Lake

**DOI:** 10.3390/toxins14020103

**Published:** 2022-01-28

**Authors:** Nur Hanisah bte Sukarji, Yiliang He, Shu Harn Te, Karina Yew-Hoong Gin

**Affiliations:** 1 NUS Environmental Research Institute (NERI), National University of Singapore, Singapore 138602, Singapore; erinhs@nus.edu.sg (N.H.b.S.); eritsh@nus.edu.sg (S.H.T.); 2 School of Environmental Science and Engineering, Shanghai Jiao Tong University, Shanghai 200240, China; ylhe@sjtu.edu.cn; 3 Department of Civil and Environmental Engineering, National University of Singapore, Singapore 117576, Singapore

**Keywords:** microcystin, *Microcystis*, mechanistic model, nitrogen, temperature, phosphorus, decay

## Abstract

Microcystin is an algal toxin that is commonly found in eutrophic freshwaters throughout the world. Many studies have been conducted to elucidate the factors affecting its production, but few studies have attempted mechanistic models of its production to aid water managers in predicting its occurrence. Here, a mechanistic model was developed based on microcystin production by *Microcystis* spp. under laboratory culture and ambient field conditions. The model was built on STELLA, a dynamic modelling software, and is based on constitutive cell quota that varies with nitrogen, phosphorus, and temperature. In addition to these factors, varying the decay rate of microcystin according to its proportion in the intracellular and extracellular phase was important for the model’s performance. With all these effects, the model predicted most of the observations with a model efficiency that was >0.72 and >0.45 for the lab and field conditions respectively. However, some large discrepancies were observed. These may have arisen from the non-constitutive microcystin production that appear to have a precondition of nitrogen abundance. Another reason for the large root mean square error is that cell quota is affected by factors differently between strains.

## 1. Introduction

Cyanobacteria blooms are becoming more prevalent throughout the world [1], often bringing a myriad of problems such as cyanotoxins and off-flavours. Microcystin is a cyclic hepatotoxin produced by many genera of cyanobacteria, including *Microcystis*, *Dolichospermum*, *Planktothrix*, *Anabaena*, *Nostoc* and *Oscillatoria* [2,3], although the most common producer cited is *Microcystis* [3,4]. Microcystin has been documented to have adverse effects on humans [5], animals [5], and even plants [5,6]. As such, the World Health Organisation has recommended a provisional guideline value of 1 μg L^−1^ for drinking water [7] for its most prevalent variant (microcystin-LR), out of the over 240 microcystin variants that have been reported [8]. In addition to its toxicity, microcystin production has been recorded across the globe, including in New Zealand [4], North America [9,10,11,12,13], China [14], Korea [15], Japan [16], Philippines [17] and Singapore [18].

Due to its toxicity and its ubiquitous nature, much research has been conducted to understand its production means and purpose. These studies have cited important effects of nitrogen [12,19], temperature [15], irradiation [20], phosphorus [21] and sulfate [22] and proposed many roles of microcystin including oxidative stress protection, adaption to environmental conditions such as low inorganic carbon, detoxification, storage of metals and communication with other cells [3]. 

Ideally, these findings will give water agencies an understanding of the potential of harmful algal blooms in their water bodies and the associated microcystin production, including when and where they would occur. Yet, in literature, most prediction models for microcystin are empirical in nature [9,10,11,23,24,25,26,27] compared to mechanistic [28,29,30]. This means that most models are localised to a specific lake and are usually non-transferable. As microcystin blooms are pervasive around the world, it would be more ideal to have a mechanistic model that is applicable across regions and that would also help understand and simulate the science of its production and degradation.

The current mechanistic models can be categorised into two types. The first is where microcystin in a newly grown cell can be estimated empirically from its growth rate [30]. This model was originally tested under nitrogen-limited conditions but found not to hold under phosphorus and irradiation varying conditions [31]. The second is a model whereby microcystin production is from new cells carrying microcystin that can be estimated via a linear regression of multiple environmental parameters [29]. While slightly statistical in nature, the model provides the flexibility of incorporating various important factors affecting microcystin cell quota. To date, this model has only been applied to lab-based cultures [28,29].

To expand the studies of mechanistic microcystin models, this study aims to build and expand on the model by Jähnichen et al. [29] to predict microcystin concentrations from (1) batch-experiments of three different strains of *Microcystis*, *M. ichthyoblabe* LP, *M. ichthyoblabe* TG, and *M. flos-aquae* LP under various temperature, nitrogen and phosphorus conditions and (2) field observations in a tropical lake. The model is first modified to account for the effects of nitrogen in addition to phosphorus and temperature, which were already included in the original model. In modelling the field data, the model is further modified by varying microcystin decay rates according to the proportion of intracellular and extracellular microcystin.

## 2. Results

### 2.1. Population in Batch Experiments

**Figure 1:**
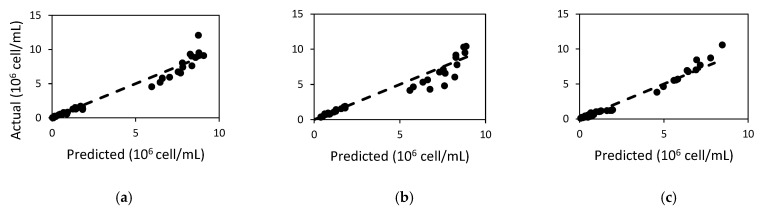
Plot of actual against predicted cell population for (**a**) *M. ichthyoblabe* LP; (**b**) *M. ichthyoblabe* TG; and (**c**) *M. flos-aquae* LP.

The model for the *Microcystis* population was able to predict the observed data well (R^2^ > 0.9 and model efficiency, MEFF > 0.9, Table 1), and especially for the nutrient experiments. The values used for the parameters in the growth model for all strains were within or close to the range found in literature for other *Microcystis* species (Table 2). As seen in the table, both *M. ichthyoblabe* strains were well fitted with the same calibration values for the population growth model, although they were isolated from different lakes. However, from Figure 1, the model is especially much better performing at low cell counts (approximately less than 2.0 × 10^6^ cell mL^−1^), with the data points being close to the 1:1 line. These low cell count observations correspond to cell counts from the varying nutrient concentrations experiment. The higher cell counts (above 4.0 × 10^6^ cell mL^−1^) correspond to the population observed in the temperature experiments, with higher nutrients. The root mean square error (RMSE) is higher for the population model in the temperature experiment than the nutrient experiment, with RMSE being about twice more for *M. flos-aquae* LP and an order of magnitude higher for both *M. ichthyoblabe* strains. It is also noticeable from Figure 1 that the model tends to underpredict at the lower end of the high cell count (between 4 × 10^6^ to 7 × 10^6^ cell mL^−1^) and overpredict when the cell count gets higher. Nevertheless, the predictions of cell density in the temperature experiment still fell within 15% percentage relative error (PRE) and was sufficient for the test of the microcystin model.

### 2.2. Microcystin Model in Batch Experiments

After the calibration in the population model, the microcystin model could be tested. The parameter values for the microcystin production intercept $p_{i}$ was dissimilar from the value used by Jähnichen et al. [29], as seen from Table 2. For this study’s, *M. ichthyoblabe* and *M. flos-aquae* strains, the cell quota calculated by the model was in the range of 0–17.3 fg cell^−1^.

#### 2.2.1. Model Performance with Varying Temperature

**Figure 2:**
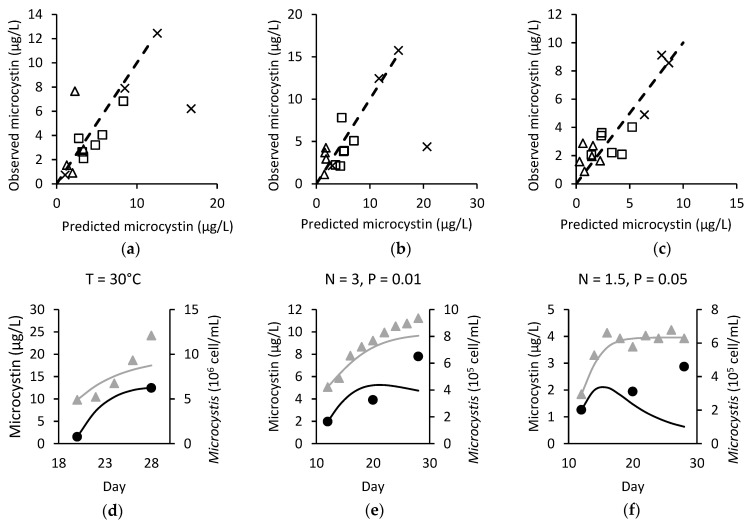
Observed against predicted microcystin for (**a**) *M. ichthyoblabe* LP; (**b**) *M. ichthyoblabe* TG; (**c**) *M. flos-aquae* LP. In (**a**–**c**), × are model points under various temperature conditions, ∆ are models points under various phosphorus conditions when nitrogen = 1.5 mg L^−1^ and □ models points under various phosphorus conditions when nitrogen = 3 mg L^−1^. Dashed line represents the 1:1 line. The modelled (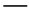) and observed (●/▲) microcystin (black) and *Microcystis* (grey) over time for (**d**) *M. ichthyoblabe* LP at 30 °C; (**e**) *M. ichthyoblabe* TG at N = 3 mg L^−1^ and P = 0.01 mg L^−1^ and; (**f**) *M. flos-aquae* LP at N = 1.5 mg L^−1^ and P = 0.05 mg L^−1^.

The calibrated value of $s_{T}$, the coefficient of temperature in microcystin cell quota, for *M. flos-aquae* LP was best suited as a positive relation (Table 2), and the model performed relatively well with RMSE, MEFF, PRE and R^2^ at 0.359, 0.751, −7.88% and 0.878 respectively. On the other hand, for both *M. ichthyoblabe* strains, although $s_{T}$ was chosen as a negative value, results indicated that the effect of temperature was not necessarily best represented as a negative linear correlation with cell quota. From Figure 2a,b, it is seen that some predictions of strains, *M. ichthyoblabe* LP and *M. ichthyoblabe* TG were off. Due to this, MEFF was negative and R^2^ < 0.367, although PRE values were 12.84%, 34.54% for the two strains respectively. Upon closer inspection, a major source of poor model performance in these two strains was due to predictions made at 27 °C conditions. With the linear model structure, lower temperature would result in higher cell quota. However, for both *M. ichthyoblabe* strains in this study, total microcystin concentration and cell quota was lower at 27 °C compared to 30 °C and 33 °C. This resulted in consistently large overestimation of microcystin concentration at the temperature 27 °C for both *M. ichthyoblabe* strains. The predicted microcystin concentration was 15.26 and 20.77 µg L^−1^, compared with the observed 6.21 and 4.38 µg L^−1^ for strains *M. ichthyoblabe* LP and TG, respectively. If predictions at 27 °C are excluded, the model performance for both *M. ichthyoblabe* drastically improved with RMSE, MEFF, PRE and R^2^ at 0.480, 0.719, 2.62%, 0.664 and 0.601, 0.694, 8.40% and 0.721 for LP and TG strain respectively.

#### 2.2.2. Model Performance with Various Nutrient Concentrations

**Figure 3:**
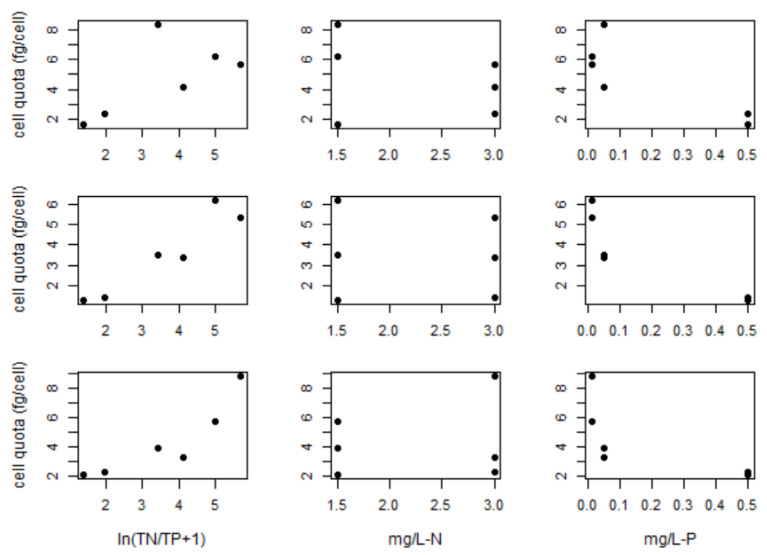
Relations between microcystin cell quota and nitrogen and phosphorus for strains *M. ichthyoblabe* LP (**top row**), *M. ichthyoblabe* TG (**centre row**) and *M. flos-aquae* LP (**bottom row**).

The varying nutrient experiments for all three strains were tested against the original and modified model and there were performance differences in RMSE, MEFF and R^2^. RMSE was always lower with the modified model (average difference 0.09) and MEFF and R^2^ is always higher in the modified model (average differences were 0.45 and 0.23 respectively). Furthermore, when comparing the calculated cell quota of each strain in the various nutrient conditions, there was no clear relation between cell quota and total nitrogen (TN). The relation with nitrogen only becomes more apparent when combining with total phosphorus (TP) (Figure 3), which by TP alone had a negative correlation as predicted by the original model [29]. Thus, it can be said that the modified model is more suitable than the original model.

With the model structure of a constitutive microcystin cell quota, it is expected that the cultures would minimally or not produce at all any microcystin once the cultures reached stationary phase (between day 20 to day 28 for most of the nutrient experiments; varying temperature experiments did not reach stationary phase). However, this was not the case for some of the culture conditions for all three strains (see Figure 2f). In cases involving *M. ichthyoblabe* LP and *M. flos-aquae* LP, the increases in microcystin concentration varied by 0.642 to 0.926 µg L^−1^ (*p*-value <0.05, paired single *t*-test) even though the cells had decreased (*p*-value < 0.05) or stayed the same. Two other cases had no statistically significant change in microcystin concentration, although it was noted that the microcystin increase was as much as 4.78 µg L^−1^ in one condition (*M. ichthyoblabe* LP, N = 1.5, P = 0.05) despite the cell concentration remaining the same. An example of the continuous microcystin production is shown in Figure 2f.

### 2.3. Microcystin Model in the Field

The model was subsequently applied to whole *Microcystis* population at three different sites, A, B and C, of a tropical lake. The range of total microcystin cell quota for *Microcystis* varied from 0.0545 to 103 fg cell^−1^ in the field (mean: 5.67 ± 15.6 fg cell^−1^). However, only Site C recorded a cell quota of 103 fg cell^−1^ on day 90 and thus was considered an anomaly. The rest of the observations were <15.5 fg cell^−1^, which was comparable to the range measured in the batch experiment. There was also no difference in average total microcystin cell quota between each site (ANOVA, *p* > 0.05).

**Figure 4:**
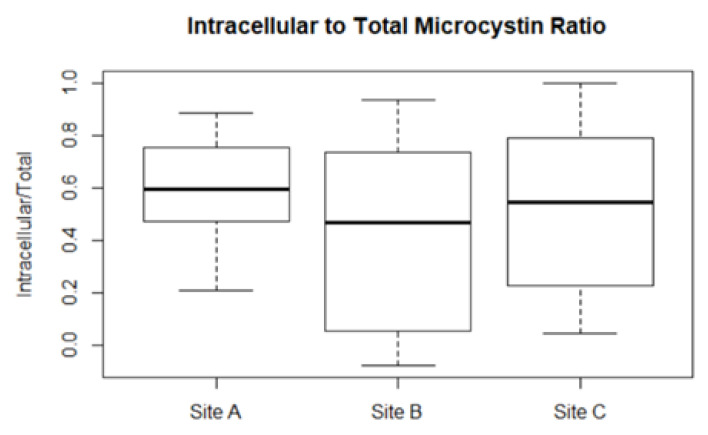
Boxplot of intracellular to total microcystin ratio at the different sites.

In the field, microcystin decay rates are much higher [37]. Hence, the decay rate was initially increased to 0.623 day^−1^, but model performance had little improvement. A possible missing factor identified for the model was that the intracellular to extracellular microcystin ratio varies throughout the sampling period, and a boxplot of its variation at different sites is given in Figure 4. When the decay rate varied throughout the simulation according to the intracellular and extracellular microcystin ratio, the model’s R^2^ improved. The improvement in model performance with varying decay rates can be seen in Table 3 for Sites A and B, but not for site C.

**Figure 5:**
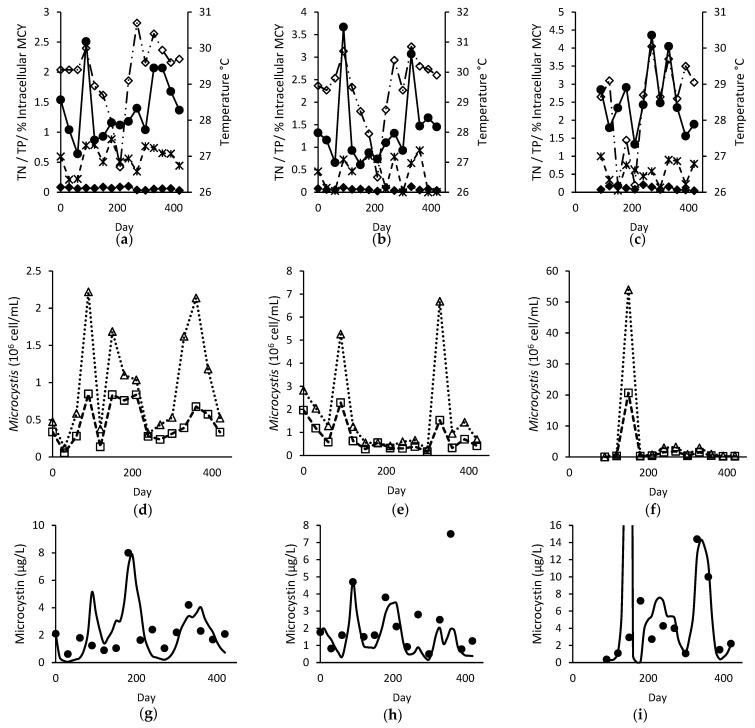
Time-varying plot of (**a**–**c**) TN (mg L^−1^) 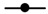, TP (mg L^−1^) 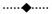, intracellular proportion of microcystin (MCY) 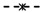 and temperature (°C) 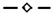; (**d**–**f**) observed total 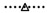 and toxigenic 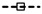 *Microcystis* population; and (**g**–**i**) modelled 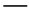 and observed ● total microcystin for Site A (left), B (middle), and C (right).

A major source of error from Site C was the microcystin prediction at day 150 (119.71 µg L^−1^), which is shown in Figure 5i. This large microcystin prediction resulted from the huge increase in *Microcystis* cells during the period (from 8.06 × 10^5^ cell mL^−1^ on day 120 to 5.40 × 10^7^ cell mL^−1^ on day 150), which was assumed to be growth (see Figure 5f). However, the actual microcystin concentration on day 150 remained low (2.94 µg L^−1^). While the toxic proportion of *Microcystis* on day 150 (38.3%) was not out of the ordinary for Site C (range: 28.9–55.8%, mean: 46.2 ± 7.95%), the proportion of total microcystin in particulate form was rather low (4.76% on day 150, compared to range of 4.76–100%, mean of 56.0 ± 31.8%). This meant that the microcystin may not necessarily have originated from *Microcystis* and that other factors could have been important, such as its transportation by diffusion, which was not modelled in this study. Without this erroneous prediction, all model performance indicators at Site C improved drastically and R^2^ showed the same pattern of improvement with the inclusion of a varied decay rate, as shown in Table 4.

**Figure 6:**
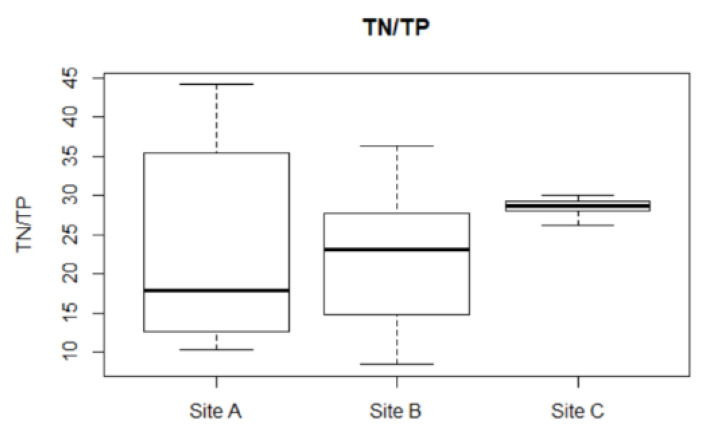
Boxplot of TN:TP at the three sites.

The model performance further improved with the inclusion of the nutrient (TN:TP) factor $s_{NP}$, as seen in the MEFF and RMSE values in Table 3 and Table 4. This is probably because TN:TP ratio varied greatly throughout the sampling period, as seen in Figure 6. According to the microcystin cell quota equation, this variation in TN:TP can result in a difference of 1.39, 1.38 and 0.13 folds in the cell quota contribution by nutrients of Sites A, B and C respectively. Although positive (implying a better performance than using the average microcystin as the prediction for all time points), MEFF was only marginally better at Sites A and B with the changes. The major source of error for Site A and Site B was the prediction on day 90 and 360 respectively. The microcystin concentration was overpredicted (5.13 µg L^−1^, compared to observed 1.24 µg L^−1^) for Site A and underpredicted (1.96 µg L^−1^ compared to observed 7.5 µg L^−1^) for Site B. Without these predictions, MEFF and R^2^ improved drastically (>0.45 and >0.55 respectively).

## 3. Discussion

The good performance of the population model in batch experiments, especially for the varying nutrient concentrations, was unsurprising as the Monod equations for nutrients have been greatly studied and used in various models [38]. There has yet to be one single equation which researchers predominantly use to describe temperature and light effects on cyanobacteria growth. Other equations such as Bernard and Rémond’s [39] cardinal temperature model and light limitation model generally have similar characteristics (i.e., increasing growth rate with increasing irradiation and temperature up to an optimum point, and a decreased growth rate past the optimum) as the one used in this study. However, a slightly poorer model performance was observed at high cell densities. This was the result of the lowered growth rates after the initial growth spurt in the temperature experiment. The calculated maximum net growth rates in the varying temperature experiment had reduced to <0.19 day^−1^ for all three strains between days 20 to 28, but was initially much higher (>0.4 day^−1^) prior to day 20 (not modelled). This lowering of growth rate could be a result of a lower nutrient concentration, dissolved inorganic carbon limitation or self-shading [40]. Self-shading was accounted for in the model, but not the varying substrate concentrations.

*Microcystis* spp. have a large variation in toxicity, from non-toxic strains to highly toxic strains with cell quotas in orders of magnitude picogram cell^−1^ [22,41]. In this study, the modelled microcystin cell quota was within the range reported for other *M. aeruginosa*, i.e., 0–30 fg cell^−1^ [42,43], but was lower than the *M. aeruginosa* in the study by Jähnichen et al., which also had a large variation in cell quota (50–150 fg cell^−1^) [29].

In most literature, temperature is negatively correlated with microcystin cell quota [15,28,29,44]. However, in the batch experiments, the *Microcystis* spp. had different relationships between cell quota and temperature. This could be because of strain differences. In this study, the *Microcystis* were species *M. ichthyoblabe* and *M. flos-aquae*, whereas in previous studies mentioned, the main species was *M. aeruginosa*. Differences in strain responses to temperature have also been observed elsewhere, where one *M. aeruginosa* strain showed a negative relationship between temperature and cell quota whereas another *M. aeruginosa* strain had lowest microcystin cell quota at 25 °C and higher at 20 and 30 °C [44]. A second reason which could explain the differences is that some factors have yet to be accounted for in the model, such as synergistic effects of environmental conditions. Song et al. [14] found that effects of temperature on microcystin production was dependent on the irradiation. Dependence between temperature and pH on microcystin production was also found by Geada et al. [45]. More research could be done on the synergistic effects to elucidate how to best model these effects. However, due to the linear general structure of the microcystin model, it could be easily modified to accommodate the different relationships.

This increases in microcystin concentration observed after the exponential growth phase in the nutrient experiments suggest that microcystin cell quota is not a constant amount for each cell at given environmental conditions and instead varies temporally. Temporal variations in microcystin cell quota have been noted in other studies [22,28,43]. Lyck [46] observed microcystin per cell was between 110 and 400 fg cell^−1^ in the exponential phase while in late stationary phase, cell quota was >400 fg cell^−1^. The observation by Lyck [46] and in this experiment could be because in the stationary phase, dying cells release intracellular contents including microcystin, which was previously shown to induce microcystin production in the resting *Microcystis* cells [47]. On contrary, in Orr and Jones’s [48] study, their axenic and non-axenic cultures did not produce more microcystin in the stationary phase, but it is noted that their cultures had depleted nitrogen by the stationary phase. In the batch experiment, the conditions in which continuous microcystin production in the stationary phase occurred in were N = 1.5 mg L^−1^, P = 0.05 mg L^−1^ (nitrogen:phosphorus; N:P molar ratio 66) for all three *Microcystis* strains, N = 1.5 mg L^−1^, P = 0.01 mg L^−1^ (N:P molar ratio 332) for *M. ichthyoblabe* TG, and N = 1.5 g L^−1^, P = 0.5 mg L^−1^ (N:P molar ratio 7) for *M. ichthyoblabe* LP. While N = 1.5 mg L^−1^, P = 0.5 mg L^−1^ conditions were not nitrogen limiting according to the Redfield ratio (molar N:P of 16; [49]), the rest were, implying there could be a minimum requirement of nitrogen before continuous microcystin production occurs. Furthermore, a recent study had suggested a modified Redfield ratio whereby cyanobacteria dominated when N:P was below 6.5 [13], which would make N = 1.5 mg L^−1^, P = 0.5 mg L^−1^ a non-nitrogen-limiting condition for cyanobacteria, which further supports this hypothesis. However, more research into this would be required.

The model performance with the field data improved greatly when decay rates varied according to the intracellular:extracellular microcystin content. This is in support of the fact that intracellular microcystin hardly degrade [50], whereas extracellular microcystin decay rates vary, depending on the availability of sediments, light, and presence of other bacteria [37]. This suggests that future microcystin models may consider the separation of the intracellular faction from the extracellular one.

While the model was able to predict most of the observations at Sites A and B, there were still some predictions that had room for improvement. The overprediction at Site A was likely due to a combination of high *Microcystis* growth (from 5.58 × 10^5^ cell mL^−1^ to 2.22 × 10^6^ cell mL^−1^), high intracellular toxin proportion (78.2%) and high TN:TP (35.9). However, the cause for the model’s large error in microcystin prediction at Site A could not be identified. The underprediction at Site B was most likely due to little growth (6.69 × 10^6^ cell mL^−1^ on day 330 to 9.57 × 10^5^ cell mL^−1^ on day 360). Both TN:TP (28.1) and intracellular portion of microcystin was high (93.3%), which would have promoted microcystin accumulation. This suggests a non-constitutive production of microcystin, which was also suspected in the batch experiment modelling.

Making a new mechanistic model generally requires a lot of studies, including the elucidation of the process and the relevant factors affecting it. While the reasons why microcystin is produced remain uncertain, the producer and its relevant factors have been identified. This allowed the model to be sufficiently able to predict the microcystin concentrations under varying nutrient and temperature conditions in the lab. With the exclusion of the few points that contributed to the large errors, most of the predictions for the batch experiments had R^2^ in the range of 0.681–0.837 (n = 43), which are comparable to the R^2^ obtained by Jähnichen et al. [29] (R^2^: 0.61–0.99) using the same model structure on other variables.

In the field, multiple phytoplankton species coexist simultaneously, including toxic and non-toxic *Microcystis*. This might have been a concern in modelling microcystin produced by *Microcystis* as shifts in their population proportion may cause unexpected spikes or absences of microcystin. This is one of the first few studies of its kind to test a mechanistic microcystin model in the field. In this study, *Microcystis* as a whole genus was used to estimate the production of microcystin. The shifts in toxic *Microcystis* proportion throughout the period (range: 22.9–102%, mean: 52.3 ± 16.2%) did not affect the prediction quality. Perhaps the other environmental factors have accounted for the changes in *Microcystis* toxicity, but more studies are recommended to affirm this. Overall, the model performance in the field excluding a few outlier predictions (R^2^ of 0.554–0.674, n = 36) was acceptable and was also comparable to some empirical models in other studies in the field (R^2^ 0.31–0.55 0.56–0.94) [9,26]. Although the RMSE was slightly bigger in this model (0.891–2.73) compared to other models (0.32–0.50) [27], further refinements of the model including the mentioned points to investigate may improve its performance. Future studies could also try incorporating this model into existing *Microcystis*-based water quality models in the field.

## 4. Conclusions

In this study, a modified Jähnichen et al. [29] mechanistic model was applied to estimate the microcystin production by *Microcystis* spp. in various experimental nutrient and temperature conditions. These elucidated a finding that perhaps microcystin production may be non-constitutive in nitrogen replete conditions. The modified model was also applied to model microcystin production by whole *Microcystis* genus in field conditions and found that variation in toxic and non-toxic strain proportion did not significantly affect the prediction, but intracellular to extracellular microcystin proportion did. Overall, the model was able to better predict most of the observations, with a MEFF of >0.726 and >0.454 respectively. However, more could be done to improve the model’s RMSE that were >0.376. These refinements could include non-constitutive cell production and non-linear relations between cell quota and temperature.

## 5. Materials and Methods

### 5.1. Data

#### 5.1.1. Batch Experiments

The data for modelling was taken from the batch experiments conducted by Mowe et al. [51,52] for three *Microcystis* strains, namely *M. ichthyoblabe* (LP20121219MI1), *M. ichthyoblabe* (TG20121219MI1), and *M. flos-aquae* (LP20121219MF1), referred to as *M. ichthyoblabe* LP, *M. ichthyoblabe* TG, and *M. flos-aquae* LP respectively in the paper. The experiments had various initial starting nutrient concentrations and varying temperature and irradiation conditions. Table 5 shows a summary of the experimental conditions used in their study. The data for growth was taken every two days, between days 8 to 28. Cell density was measured with OD_680_, which was linearly calibrated against cell density counted with a Sedgwick Rafter counting chamber and compound microscope (R^2^ > 0.9 and *p*-value < 0.05 for all strains for different stages of growth).

Microcystin (MC) data was only available on days 12, 20 and 28 in the varying nutrient experiment (constant light and temperature across cultures) and days 20 and 28 in the varying temperature (constant nutrient and light across cultures) experiment. In the varying temperature experiment, both intracellular and extracellular MC-LR and MC-RR were analysed. However, for the varying nutrient experiments, MC-LR was only detected in a few samples and there was no extracellular data. Hence, varying nutrient experimental data consisted primarily of intracellular MC-RR. All microcystin measurements for the batch experiment were freeze-dried then extracted twice with 75% methanol and 25% water mixture. The analysis was carried out using LC-MS/MS. More details can be found in [51,52]. For the model, the first available data was used as the initial values (either data on day 12 or day 20) and was not counted in the model performance. Only results from the subsequent measurement day(s) with data were compared against the prediction.

#### 5.1.2. Field Data

The field data was taken from [18] which studied a eutrophic lake in Singapore. The catchment comprises a mix of urban (business and residential) and undeveloped (agricultural, open spaces, cemeteries etc.) land use in approximately the proportion of 35% and 65%, respectively. The average depth of the lake is 3.5 m and the maximum depth is 17 m. The general statistics of the general water quality parameters of this lake are given in Table 6.

In this study, *Microcystis* was identified as the main producer of microcystin [18]. There were three sites studied within the lake, which included one site at the merger of all tributaries of the lake (Site A), one site in a deep part of the lake (Site B), and one site in a tributary (Site C). The sampling period was from February 2008 to August 2009. However, some microcystin data were missing and hence the modelling only began from June 2008 for Sites A and B, and from September 2008 for Site C. Each month in the model was taken as 30 days and June 2008 was taken as day 0.

The available data from the sampling included intracellular and extracellular microcystin, temperature, toxigenic and non-toxigenic *Microcystis* spp. cell count. These were measured using ELISA assay, YSI meter, qPCR of *Microcystis*-specific 16S rRNA gene and mcy gene respectively. All measurements for the field data are detailed in [18]. However, there were no dissolved inorganic nitrogen (DIN), dissolved inorganic phosphorus (DIP) and irradiation data available. Thus, it was difficult to model the growth of *Microcystis* and therefore was not conducted. However, *Microcystis* growth rate was still required for the microcystin model. The growth of *Microcystis* was back calculated from the data with mortality accounted for, following the equation below:

(1) $$2*\left(\frac{\ln\left(\frac{H_{t}}{H_{t-dt}}\right)}{dt}+R\right)$$

where $H_{t}$ is the *Microcystis* spp. cell concentration at time t, $H_{t-dt}$ is the *Microcystis* spp. cell concentration at time t−dt, dt is the time interval between samplings (taken as 30 days to represent one month) and R is the respiration and mortality rate (taken as a constant of 0.15 day^−1^). The equation is multiplied by two to account for the day-night cycle. The *Microcystis* spp. used for this calculation included both toxigenic and non-toxigenic strains.

### 5.2. Model Platform

Modelling was done on Systems Thinking Experimental Learning Laboratory with Animation (STELLA) by iseesystems, a visual dynamic modelling software. It is capable of modelling various model structures with its building blocks (stocks, flows, converters and connectors) and also has flexible equations input. In this study, the Runge-Kutta 4th order method was used. Preliminary testing revealed that model simulation results converged around an interval of 0.1 day which was subsequently used for all runs.

### 5.3. Model Equations

In this model, microcystin is produced when new *Microcystis* cells are grown, with each new cell containing a value of microcystin known as the cell quota. The microcystin cell quota is affected by temperature, nitrogen and phosphorus. Equations for these are given below in Table 7. *Microcystis* cell growth in the lake was detailed earlier, while in the batch experiments, the growth of the *Microcystis* cells was modelled following simple Monod kinetics, which can be limited by light, temperature, DIN or DIP. The *Microcystis* population decreases by mortality. Equations used for this portion are shown in Table 8.

It is noted that some of the relationships between microcystin cell quota and the various environmental variables in [29] are not used here, such as relations with log(Fe^3+^) and SO_4_^2−^. This is because of the lack of variation in concentration during the batch experiments, implying its impact could be accounted for as a constant. In the field, these parameters were not measured and hence could not be modelled. Also, In the original model in [29], only phosphate was related to cell quota. However, many other papers have cited the positive correlation between nitrogen concentration and toxin production of *Microcystis* [31,55]. This correlation also makes sense stoichiometrically as microcystin is a nitrogen-rich compound. However, as [12] mentioned that the cell quota was affected by nitrogen in relation to phosphorus, the overall modification of the model was replacing the phosphorus factor with a nitrogen-phosphorus factor, which was related to TN/TP. A varying decay rate was also tested on the lake according to the intracellular and extracellular proportion of microcystin. This ratio was calculated from the data available from the sampling. Decay rates in between sampling points were extrapolated.

### 5.4. Model Performance

Calibration of the parameters was performed manually by changing one parameter at a time, for parameters of *Microcystis* growth and microcystin production. Model fit was examined qualitatively by visual inspection and quantitatively by root mean square error (RMSE), coefficient of determination (R^2^), percentage relative error (PRE), model efficiency (MEFF). The equations for the model performance indicators are given below:

(2) $$RMSE=\sqrt{\frac{\sum_{i=1}^{N}{\left(P_{i}-O_{i}\right)}^{2}}{N}}$$

(3) $$R^{2}={\left(\frac{\sum_{i=1}^{N}\left(P_{i}-\bar{P}\right)\left(O_{i}-\bar{O}\right)}{{\left[\sum_{i=1}^{N}{\left(P_{i}-\bar{P}\right)}^{2}\sum_{i=1}^{N}{\left(O_{i}-\bar{O}\right)}^{2}\right]}^{\frac{1}{2}}}\right)}^{2}$$

(4) $$PRE=\frac{\sum_{i=1}^{N}\frac{\left(P_{i}-O_{i}\right)}{O_{i}}}{N}*100$$

(5) $$MEFF=1-\frac{\sum_{i=1}^{N}{\left(P_{i}-O_{i}\right)}^{2}}{\sum_{i=1}^{N}{\left(O_{i}-\bar{O}\right)}^{2}}$$

where $P_{i}$ is the predicted ith data point, $O_{i}$ is the observed ith data point, $\bar{P}$ is the average predicted value, $\bar{O}$ is the average observed value, and N is the number of data points.

## Figures and Tables

**Table 1:** Root mean square error (RMSE), model efficiency (MEFF), percentage relative error (PRE) and coefficient of determination (R^2^) for *Microcystis* cell counts.

*Microcystis* Strain	RMSE (×10^5^)	MEFF	PRE	R^2^
*M. ichthyoblabe* LP	3.62	0.964	12.2	0.982
*M. ichthyoblabe* TG	4.31	0.934	1.12	0.970
*M. flos-aquae* LP	2.642	0.970	9.43	0.978

**Table 2:** Final model calibration values and range of values found from literature. Many studies reported varying values for the parameters. Only the relevant studies with the highest and lowest values for each parameter is referenced. Parameters without reported values are indicated with a dash.

Parameter (Unit)	*M. ichthyoblabe* LP	*M. ichthyoblabe* TG	*M. flos-aquae* LP	From Literature
$K_{SP}$ (mg L^−1^)	0.013	0.013	0.0063	0.00109–0.012 [32]
$K_{SN}$ (mg L^−1^)	0.19	0.19	0.1	0.008–0.53 [17,33,34]
α (d^−1^ (µmol photons m^−2^ s^−1^)^−1^)	0.06	0.06	0.025	0< (value depends on optimum light for growth, which varies between species and strain; [35,36])
µ (d^−1^)	1.81	1.81	1.8	0.493–2.2 [32,35]
−β (d^−1^ (µmol photons m^−2^ s^−1^)^−1^)	−0.00525	−0.00525	0	≤0 (value depends on photoinhibition effect, which varies between species and strains) [35,36]
$\psi_{1}$ (–)	1.4	1.4	1.309	–
b (–)	0.7	0.7	0	–
d (–)	1.0037	1.0037	1.0765	–
a (–)	20.065	20.065	21	–
$k_{m}$ (d^−1^)	0.03	0.03	0.01	–
$\psi_{2}$ (–)	1.05	1.05	1.01	–
$s_{\mathrm{NP}}$ (fg cell^−1^)	2.8	2.8	2.3	–
$s_{T}$ (fg cell^−1^ °C^−1^)	−0.55	−0.61	0.49	−2.15 [29]
$p_{i}$ (fg cell^−1^)	14.7	17.8	−14.7	177.1 [29]
$k_{d}$ (d^−1^)	0.16	0.16	0.16	0.047–0.746 [37]

**Table 3:** Model performance with different decay rates for the three sites. In the last simulation, $s_{NP}$, microcystin cell quota coefficient with total nitrogen (TN) and total phosphorus (TP), was included. Varied decay rates depend on the intracellular:extracellular microcystin.

		Site A				Site B				Site C			
$s_{NP}$, $s_{T}$, $p_{i}$	Decay Rate (day^−1^)	MEFF	R^2^	RMSE	PRE	MEFF	R^2^	RMSE	PRE	MEFF	R^2^	RMSE	PRE
0, −2.15, 66.5	0.16	−1.39	0.07	2.81	86.19	−1.05	0.06	2.65	107.07	−497.65	0.02	137.83	1497.23
	0.623	−0.62	0.04	2.31	−53.28	−0.94	0.09	2.57	−51.83	−58.31	0.02	47.54	437.87
	Varied	0.06	0.40	1.77	29.66	0.08	0.23	1.77	−16.38	−66.01	0.01	50.53	523.52
2, −2.15, 60	Varied	0.15	0.40	1.67	25.75	0.16	0.32	1.70	−23.25	−50.68	0.00	44.37	451.19

**Table 4:** Model performance with different decay rates for Site C without day 150. In the last simulation, $s_{NP}$ was included. Varied decay rates depend on the ratios between intracellular and extracellular microcystin.

		Site C without Day 150			
$s_{NP}$, $s_{T}$, $p_{i}$	Decay Rate	MEFF	R^2^	RE	PRE
0, −0.55, 21.39	0.16	0.09	0.08	6.11	93.45
	0.623	0.31	0.04	5.32	−51.54
	Varied	0.75	0.53	3.20	6.89
2, −2.15, 60	Varied	0.81	0.63	2.77	−3.36

**Table 5:** Environmental conditions in batch experiments by Mowe et al. [51,52].

Condition Name		Nitrogen (mg-N L^−1^)	Phosphorus (mg-P L^−1^)	Temperature (°C)	Irradiance (µmol m^−2^ s^−1^)	Light:Dark
Varying nutrient	N = 1.5, P = 0.01	1.5	0.01	27	45	24:0
	N = 1.5, P = 0.05		0.05			
	N = 1.5, P = 0.5		0.5			
	N = 3, P = 0.01	3	0.01			
	N = 3, P = 0.05		0.05			
	N = 3, P = 0.5		0.5			
Varying temperature	27 °C	28	6.19	27	60	12:12
	30 °C			30		
	33 °C			33		
	36 °C			36		

**Table 6:** Range and mean of water quality parameters in the lake.

Parameter	Range (Mean)	Unit
DO	1.97–14.4 (6.77)	mg L^−1^
Temperature	26.2–30.9 (29.1)	°C
pH	7.1–10.4 (8.50)	–
Conductivity	107–303 (222)	µS cm^−1^
Secchi depth	19–100 (50.2)	cm
Chlorophyll a	8.43–1585 (155)	µg L^−1^
Total organic carbon	3.12–24.6 (9.96)	mg L^−1^
Total nitrogen (TN)	0.61–4.36 (1.71)	mg L^−1^
Total phosphorus (TP)	0.0272–0.211 (0.0877)	mg L^−1^
Turbidity	5.76–225 (53.5)	NTU

**Table 7:** Equations used for microcystin production.

Description	Equation	Reference
Rate of change of total microcystin (μg L^−1^ d^−1^)	$\frac{dM}{dt}=p*\mu *H-k_{d}*M$	[29]
Microcystin production (fg cell^−1^)	$p=s_{T}*T+s_{P}*\ln\left(TP\right)+p_{i}$	Original model from [29]
Microcystin production (fg cell^−1^)	$p=\max\left(s_{T}*T+s_{\mathrm{NP}}*\ln\left(\frac{TN}{TP}+1\right)+p_{i},\;0\right)$	Modified from [29]
Microcystin decay rate (in field test)	$k_{d}=k_{dIntra}*\frac{M_{Intra}}{M}+k_{dExtra}*\frac{M_{Extra}}{M}$	

**Table 8:** *Microcystis* growth equations.

Description	Equation	Reference
Rate of change of *Microcystis* (cell d^−1^)	$\frac{dH}{dt}=\left(\mu -R\right)*H$	
Growth rate of *Microcystis* (d^−1^)	$\mu =(\mu_{max}*f\left(T\right)*\min\left(f\left(I\right),f\left(DIN\right),f\left(DIP\right)\right)-\beta I)*\left(1-\frac{H}{C}\right)$	
Temperature limitation	$f\left(T\right)=\psi_{1}{}^{T-20}-\psi_{1}{}^{d\left(T-a\right)}+b$	[33]
Light limitation	$f\left(I\right)=(1-e^{-\frac{\alpha I}{\mu_{max}}})$	[53]
Nitrogen limitation	$f\left(DIN\right)=\frac{DIN}{DIN+K_{SP}}$	[54]
Phosphorus limitation	$f\left(DIP\right)=\frac{DIP}{DIP+K_{SP}}$	[54]
*Microcystis* respiration and mortality rate (d^−1^)	$R=k_{m}*\psi_{2}^{T-20}$	
Irradiance (µmol photons m^−2^ s^−1^)	$I=\frac{I_{0}}{10^{OD}}$	

## Data Availability

Data was obtained from Dr. Mowe and Dr. Te and are available from [51,52] and from [18], respectively, with the authors’ permissions.

## Abbreviations

**Symbol**	**Description**	**Unit**
p	Microcystin cell quota	fg cell^−1^
$k_{d}$	Decay rate of microcystin	d^−1^
$s_{T}$	Temperature coefficient for microcystin cell quota	fg cell^−1^ °C^−1^
$s_{P}$	Phosphorus coefficient for microcystin cell quota	fg cell^−1^
$s_{\mathrm{NP}}$	Nutrient coefficient for microcystin cell quota	fg cell^−1^
$p_{i}$	Microcystin cell quota intercept	fg cell^−1^
TN	Total nitrogen	mg-N L^−1^
TP	Total phosphorus	mg-P L^−1^
$k_{dIntra}$	Decay rate of intracellular microcystin (0 d^−1^)	d^−1^
$k_{dExtra}$	Decay rate of extracellular microcystin (0.623 d^−1^)	d^−1^
$M_{Intra}$	Intracellular microcystin	µg L^−1^
$M_{Extra}$	Extracellular microcystin	µg L^−1^
H	Algae cells	cell mL^−1^
µ	Growth Rate	d^−1^
R	Mortality and respiration rate	d^−1^
$\mu_{max}$	Maximum specific growth rate	d^−1^
T	Water temperature	°C
I	Irradiance	µmol photons m^−2^ s^−1^
DIN	Initial dissolved inorganic nitrogen	mg-N L^−1^
DIP	Initial dissolved inorganic phosphorus (orthophosphate)	mg-P L^−1^
−β	Inhibition rate at high irradiance	d^−1^ (µmol photons m^−2^ s^−1^)^−1^
C	Carrying Capacity	cell mL^−1^
$\psi_{1}$	Temperature coefficient	—
d	Temperature coefficient	—
a	Temperature coefficient	—
b	Temperature coefficient	—
α	Growth rate increase at low irradiance	d^−1^ (µmol photons m^−2^ s^−1^)^−1^
$K_{SN}$	Nitrogen half saturation constant	mg-N L^−1^
$K_{SP}$	Phosphorus half saturation constant	mg-P L^−1^
$k_{m}$	Mortality rate at 20 °C	d^−1^
$\psi_{2}$	Temperature coefficient	—
